# An Autoimmune Disease-Associated Risk Variant in the *TNFAIP3* Gene Plays a Protective Role in Brucellosis That Is Mediated by the NF-κB Signaling Pathway

**DOI:** 10.1128/JCM.01363-17

**Published:** 2018-03-26

**Authors:** Lixin Lou, Wanguo Bao, Xianjun Liu, Hongxiao Song, Yang Wang, Kaiyu Zhang, Wenjing Gao, Haijun Li, Zhengkun Tu, Shaofeng Wang

**Affiliations:** a Department of Infectious Diseases, The First Hospital of Jilin University, Jilin, China; b The Bethune Institute of Epigenetic Medicine, The First Hospital of Jilin University, Jilin, China; c Department of Translational Medicine, The First Hospital, Jilin University, Jilin, China; Memorial Sloan Kettering Cancer Center

**Keywords:** brucellosis, inflammation, single nucleotide polymorphism, TNFAIP3, NF-κB

## Abstract

Naturally occurring functional variants (rs148314165 and rs200820567, collectively referred to as TT>A) reduce the expression of the tumor necrosis factor alpha-induced protein 3 (*TNFAIP3*) gene, a negative regulator of NF-κB signaling, and predispose individuals to autoimmune disease. In this analysis, we conducted a genetic association study of the TT>A variants in 1,209 controls and 150 patients with brucellosis, an infectious disease, and further assessed the role of the variants in brucellosis. Our data demonstrated that the TT>A variants were correlated with cases of brucellosis (*P* = 0.002; odds ratio [OR] = 0.34) and with individuals who had a positive serum agglutination test (SAT) result (titer of >1/160) (*P* = 4.2 × 10^−6^; OR = 0.23). A functional study demonstrated that brucellosis patients carrying the protective allele (A) showed significantly lower expression levels of the TNFAIP3 gene in their peripheral blood mononuclear cells and showed increased NF-κB signaling. Monocytes from individuals carrying the A allele that were stimulated with Brucella abortus had lower mRNA levels of TNFAIP3 and produced more interleukin-10 (IL-10), IL-6, and IL-1β than those from TT allele carriers. These data showed that autoimmune disease-associated risk variants, TT>A, of the TNFAIP3 locus play a protective role in the pathogenesis of brucellosis. Our findings suggest that a disruption of the normal function of the TNFAIP3 gene might serve as a therapeutic target for the treatment of brucellosis.

## INTRODUCTION

**FIG 1:**
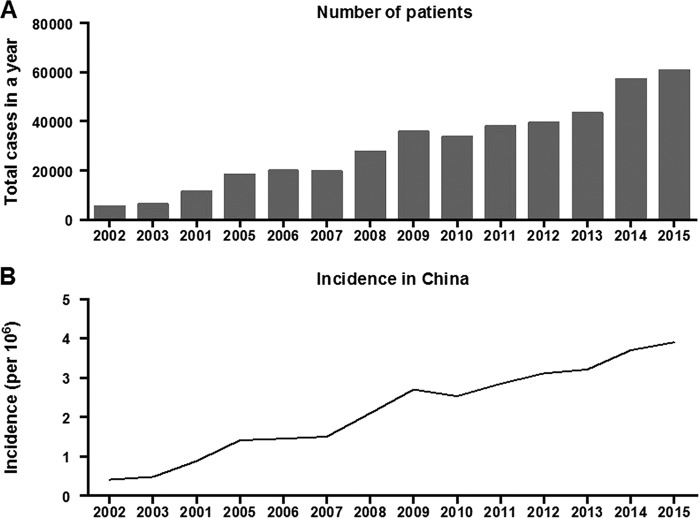
The annual incidence rate of brucellosis in China has increased from 2002 to 2015. These national data were collected from the official website of the National Bureau of Statistics of China. (A) Total number of brucellosis patients in China each year from 2002 to 2015. (B) Annual total incidence of brucellosis in China from 2002 to 2015 (incidence range, 0.41 to 4.22 per 10^6^ individuals).

Brucellosis is a zoonosis caused by Gram-negative bacteria of the genus Brucella that infect many farm animals, including cattle, sheep, goats, and pigs. There are approximately half a million human cases of Brucella infections per year, even though humans are only incidental hosts (http://data.stats.gov.cn/index.htm). There is no approved human vaccine available. In humans, brucellosis typically presents with a high, undulating fever. However, chronic brucellosis may affect multiple host organs, leading to arthritis, orchitis, encephalomyelitis, and endocarditis. Osteoarticular disease represents the most common complication (1). The diverse manifestations of brucellosis make the diagnosis of this disease even more complicated. Brucellosis in humans and livestock is relatively uncommon in industrialized nations. However, brucellosis is endemic in many developing regions, including China. As shown in Fig. 1, the annual incidence rate of brucellosis in China has increased from 2002 to 2015 (http://data.stats.gov.cn/index.htm).

The interaction of Brucella with the human immune system is critical for the development of chronic parasitism and the clearance of infection (2–4). NF-κB signaling plays a vital role in the immune system by regulating innate and adaptive immunity (5). Bacterial and viral infections rapidly induce the activation of NF-κB signaling, producing a potent inflammatory response. It is known that immune responses vary among individuals. These differences between individuals can be partly explained by the presence of genetic polymorphisms within immune-response-related genes that regulate the activities of inflammatory signaling pathways.

The tumor necrosis factor alpha-induced protein 3 (*TNFAIP3*) gene encodes the ubiquitin-editing enzyme A20, a key negative regulator of NF-κB activity. Genetic studies have suggested a role for *TNFAIP3* in susceptibility to complex genetic autoimmune disorders (6–12), including systemic lupus erythematosus (SLE) (13–24). In our previous studies, we described a pair of tandem polymorphic dinucleotides (rs148314165 and rs200820567, collectively referred to as TT>A) that decrease the gene expression level of *TNFAIP3* (25–27). Further characterization of the molecular mechanisms involved revealed that the TT>A variants reside in an enhancer element that binds the transcription factors NF-κB and SATB1, enabling the interaction of the TT>A enhancer with the *TNFAIP3* promoter through long-range DNA looping. Impairment of NF-κB binding to the TT>A risk alleles inhibits the looping interaction, resulting in reduced A20 expression. Additionally, transcription activation-like effector nuclease (TALEN)-mediated knockout of the TT>A enhancer in HEK293T cells enhanced the activity of NF-κB signaling.

Cell-mediated immunity serves a crucial role in host protection against Brucella. Among the cells responsible for this, macrophages are key elements in the cellular immune response against intracellular Brucella (28, 29). Activated macrophages use a broad range of phagocytic and inducible bactericidal functions to kill Brucella. Moreover, infected macrophages produce proinflammatory cytokines and chemokines. A previous study showed that A20 knockdown in macrophages promoted Brucella abortus-induced NF-κB activation and macrophage cell death, which suppressed B. abortus intracellular replication (30). Additionally, several regulatory molecules secreted by monocytes may contribute to the intracellular survival of Brucella (4, 31).

The TT>A variants at the *TNFAIP3* gene are important modifiers of the NF-κB signaling pathway, and we hypothesized that the TT>A variants might play a role in the pathogenesis of brucellosis. Therefore, we carried out a case-control study to estimate the association of the TT>A variants of the *TNFAIP3* locus and further assessed the role of the TT>A variants in influencing the expression of cytokines downstream of the NF-κB signaling pathway.

## MATERIALS AND METHODS

### Subjects.

The study was carried out with people of Chinese Han descent who were affected with brucellosis and were living in Jilin Province, northeast China, and with healthy animal husbandmen. The Ethical and Clinical Trial Committee of the First Hospital of Jilin University approved the study protocol. Blood samples were collected in the participants' places of residence after obtaining their informed written consent. All the patients were either farmers keeping animals (including animals that had been diagnosed with infections) or individuals who had a history of consuming raw milk and unpasteurized dairy products. Brucellosis was diagnosed according to clinical findings (e.g., fever, night sweating, weakness, malaise, weight loss, splenomegaly, myalgia, and arthralgia) and positive serological tests, defined as high titers in a standard agglutination test (SAT) (SAT titer of >1/160). The patients were selected based on the exclusion of other infectious, autoimmune, and malignant diseases. The control group was selected randomly among healthy animal husbandmen who had previous exposure to Brucella-infected animals and had consumed their milk and dairy products. They did not show any clinical manifestations after 6 months. The patients and controls were from the same geographical areas.

### ELISA.

Enzyme-linked immunosorbent assays (ELISAs) for Brucella-targeted IgG and IgM (Vircell, SL, Spain) were performed by using patient sera. ELISA kits were run and results were interpreted according to the manufacturer's procedures and recommendations.

### DNA collection and genetic polymorphism analysis.

Blood samples (∼2 ml) were stored at −80°C, thawed in batches of 48, and centrifuged to obtain cellular pellets. DNA was isolated from these pellets (200 μl) with DNA extraction kits (TransGen Biotech, Beijing, China). DNA concentrations were determined with the aid of a Synergy H1 Hybrid Multi-Mode microplate reader (BioTek, VT, USA), with purity being estimated by measuring the ratio of the optical density at 260 (OD_260_) to the OD_280_.

### SNP genotyping.

The analysis of genetic polymorphisms was performed by using quantitative real-time PCR (qPCR) with a TaqMan probe. The single nucleotide polymorphism (SNP) rs7749323 in the A20 gene was genotyped by using predesigned TaqMan SNP genotyping assays (Thermo Scientific, DE, USA). The sequences of the primers and of the probe for TaqMan genotyping are shown in Table S3 in the supplemental material. Amplifications were performed with an Agilent Stratagene Mx3005P real-time PCR system (Agilent Technologies, USA) using 96-well plates.

### Studies of *TNFAIP3*, IL-10, IL-6, and IL-1β.

Peripheral blood mononuclear cells (PBMCs) were isolated and purified by density gradient centrifugation using lymphocyte separation medium (Fresenius Kabi Norge AS, Halden, Norway). CD14^+^ cells were isolated and purified by using CD14 microbeads (Miltenyi Biotec, Inc., Auburn, CA, USA). The purities of the CD14^+^ cells were determined by flow cytometry (BD LSRFortessa cell analyzer; BD Biosciences, NJ, USA), and the purity was always >90%.

CD14^+^ monocytes were cultured in the presence of 1.0 μg/ml smooth lipopolysaccharides (S-LPS) from Escherichia coli (Sigma-Aldrich, Inc., St. Louis, MO, USA), as previously described (32–34), or in the presence of heat-killed B. abortus (multiplicity of infection [MOI] of 100:1) for 12 h. mRNAs of the stimulated or control cells were then isolated and subjected to gene expression analyses. In brief, mRNAs were purified with an Easypure RNA kit (TransGen Biotech, Inc., Beijing, China) and reverse transcribed into cDNA according to the manufacturer's instructions. The cDNAs were amplified by qPCR with a real-time PCR system (Applied Biosystems StepOnePlus; Life Technologies, CA, USA). The target gene expression level was determined by using the comparative cycle threshold (ΔΔ*C_T_*) method and normalized to the value for glyceraldehyde-3-phosphate dehydrogenase (GAPDH). The primers are shown in Table S4 in the supplemental material.

The protein expression levels of interleukin-10 (IL-10), IL-6, and IL-1β in monocytes were determined by using phycoerythrin (PE)-conjugated anti-human IL-10, allophycocyanin (APC)-conjugated anti-human IL-6, and PE-conjugated anti-human IL-1β in separate reactions analyzed by using the BD LSRFortessa instrument after intracellular staining with a fixation/permeabilization solution kit (BD) according to the manufacturer's instructions. All the antibodies were obtained from BD Biosciences. The data acquired were analyzed with FlowJo software (TreeStar Software, Ashland, OR, USA).

### Statistical analysis.

Single-marker associations were assessed by using the logistic regression function in Plink, version 1.09. The *P* value for the Hardy-Weinberg proportion test of the rs7749323 variant in controls was >0.01. The odds ratios (ORs) and 95% confidence intervals (CIs) were also calculated. The ORs and 95% CIs were adjusted according to the age and sex of the individual. All analyses were two tailed, and differences were interpreted as statistically significant when the *P* value was <0.05.

## RESULTS

### Characteristics of samples in this study.

In total, 221 SAT-positive individuals were recruited, including 71 subjects devoid of any clinical symptoms of brucellosis and 150 brucellosis patients. All patients with brucellosis were diagnosed according to clinical findings, including arthralgia, fever, sweating, hepatomegaly, splenomegaly, focal complication, and the presence of high titers of specific antibodies. According to the ELISA results, the patients were divided into IgM-positive (*n* = 136) and IgG-positive (*n* = 120) groups. An additional 1,209 matched population controls were enrolled in the same geographic location. Arthralgia, fever, fatigue, and sweating were common in most of the patients. Other relevant patient clinical characteristics at presentation are listed in Table 1.

**TABLE 1:** Genetic variant rs7749323 at the TNFAIP3 locus associated with brucellosis and SAT-positive individuals^*a*^

Parameter	No. of samples	No. of samples of genotype			MAF (A allele)	*P*	OR (95% CI)
		AA	AG	GG			
Controls	1,209	8	144	1,057	0.06617		
Cases	150	0	7	143	0.02333	2.0 × 10^−3^	0.3372 (0.1566–0.7257)
Laboratory tests							
ELISA results for patients							
IgM^+^	136	0	6	130	0.02206	2.1 × 10^−3^	0.3183 (0.1395–0.7263)
IgG^+^	120	0	5	115	0.02083	2.9 × 10^−3^	0.3003 (0.1221–0.7387)
PAT^+^ and SAT titer of >1/160	221	0	7	214	0.01584	4.2 × 10^−6^	0.2271 (0.1058–0.4874)
Characteristics of patients							
Gender							
Male	105	0	4	101	0.01905	4.2 × 10^−3^	0.2741 (0.1006–0.7466)
Female	45	0	3	42	0.03333	0.2777	0.4866 (0.1522–1.556)
Fever							
≥37.3°C	112	0	6	106	0.02679	0.02006	0.3884 (0.1699–0.8879)
<37.3°C	38	0	1	37	0.01316	0.09014	0.1882 (0.02599–1.362)
Cold							
Positive	26	0	1	25	0.01923	0.2551	0.2767 (0.03799–2.015)
Negative	124	0	6	118	0.02419	5.6 × 10^−3^	0.3499 (0.1532–0.799)
Sweating							
Positive	72	0	4	68	0.02778	0.07763	0.4032 (0.1473–1.103)
Negative	78	0	3	75	0.01923	0.01624	0.2767 (0.08728–0.8773)
Fatigue							
Positive	84	0	5	79	0.02976	0.07066	0.4329 (0.1753–1.069)
Negative	66	0	2	64	0.01515	0.01551	0.2171 (0.05324–0.8855)
Chilly							
Positive	9	0	0	9	0	0.6264	NA
Negative	141	0	7	134	0.02482	3.9 × 10^−3^	0.3592 (0.1668–0.7736)
Arthralgia							
Positive	108	0	5	103	0.02315	8.1 × 10^−3^	0.3344 (0.1358–0.8235)
Negative	42	0	2	40	0.02381	0.1712	0.3442 (0.08388–1.412)
Headache							
Positive	30	0	1	29	0.01667	0.1799	0.2392 (0.03293–1.738)
Negative	120	0	6	114	0.025	0.01093	0.3619 (0.1584–0.8266)
Lumbago							
Positive	59	0	2	57	0.01695	0.03152	0.2433 (0.05959–0.9936)
Negative	91	0	5	86	0.02747	0.03911	0.3987 (0.1616–0.9836)
Orchitis							
Positive	2	0	1	1	0.25	0.2407	4.704 (0.4866–45.48)
Negative	148	0	6	142	0.02027	7.5 × 10^−4^	0.292 (0.1281–0.6657)

a PAT, plate agglutination test; MAF, minor allele frequency; NA, not applicable.

### The rs7749323 variant (G>A) is a perfect proxy for the TT>A polymorphism downstream of the *TNFAIP3* gene in a Chinese population.

**FIG 2:**
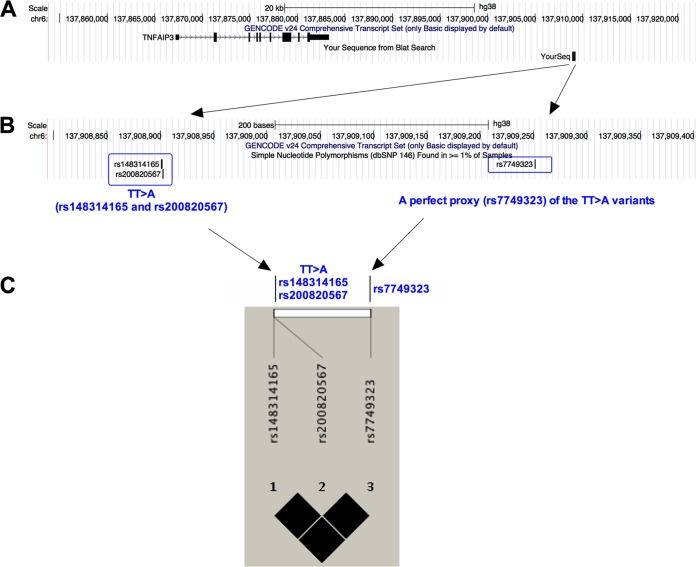
Variant rs7749323 is a perfect proxy for the functional TT>A variants downstream of the *TNFAIP3* gene in samples from Chinese individuals. (A) We used the table browser tool in the UCSC Genome Browser to show the relative locations of the TT>A variants, proxy variant rs7749323, and the *TNFAIP3* gene. (B) Magnified view of the region containing the TT>A variants and the proxy variant. (C) Plot of the pairwise linkage disequilibrium of the three variants with the color for *r*^2^ superimposed.

The TT>A variants each include a deletion of T followed by a T-to-A transversion at positions 138272732 and 138271733 on chromosome 6, 42 kb downstream from the *TNFAIP3* gene. To obtain accurate genotypes of the TT>A variants (which include both an SNP and a deletion), a perfect proxy SNP (rs7749323) of the TT>A variants was selected and genotyped in our cohort. The proxy SNP is in complete linkage disequilibrium (LD) with the TT>A variants (*r*^2^ = 1) in European and Korean individuals (25, 27). To assess the frequency of the TT>A variants in our cohort, we resequenced an 850-bp DNA fragment, centered on the TT>A variants, in 50 brucellosis cases and 50 healthy controls. As shown in Fig. 2 and Table S1 in the supplemental material, the TT>A variants are in complete LD with the rs7749323 variant, with an *r*^2^ value of 1.

### The SLE-associated risk allele (T) plays a protective role in brucellosis.

The demographics of 150 brucellosis cases and 1,209 matched population controls enrolled in the study are shown in Table S2 in the supplemental material. There were no significant differences between patients and control subjects in terms of mean age or gender distribution. Single-marker association was performed by using logistic regression. We observed a significant negative association (*P* = 2.0 × 10^−3^; OR = 0.34) between the minor A allele of the rs7749323 variant and brucellosis in our cohort, indicating that this variant plays a protective role (Table 1). Next, we assessed the role of rs7749323 in 221 SAT-positive samples; interestingly, we observed a stronger association of rs7749323 (*P* = 4.2 × 10^−6^; OR = 0.23) with SAT positivity than with the occurrence of brucellosis, suggesting a more important role of the variant in SAT-positive samples. We further assessed the genetic association between variant rs7749323 and IgM- or IgG-positive patients with brucellosis. As shown in Table 1, we observed significant associations of the variant with both subgroups (*P* = 0.0029 for IgG-positive patients and *P* = 0.0021 for IgM-positive patients). To further evaluate the role of rs7749323 in brucellosis, we stratified cases based on clinical phenotypes. As shown in Table 1, we found that the rs7749323 polymorphism was associated with multiple clinical phenotypes of brucellosis.

### rs7749323 is associated with reduced expression of the *TNFAIP3* gene, decreased sensitivity to stimulations with B. abortus, and increased expression of NF-κB1.

**FIG 3:**
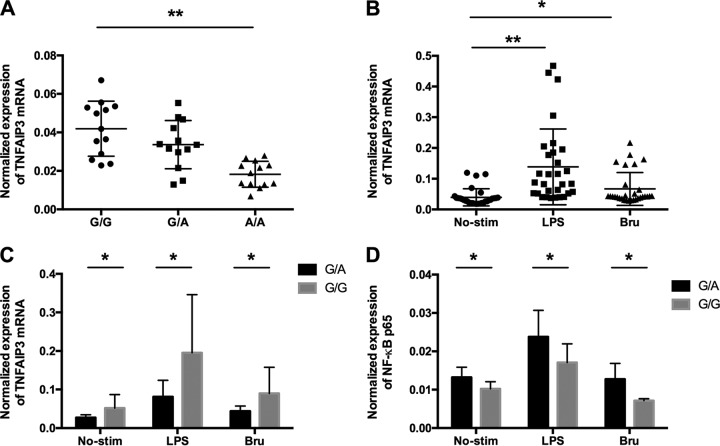
Protective allele A reduces mRNA expression levels of *TNFAIP3* in PBMCs and monocytes. (A) The three different genotypes for the proxy variant are displayed on the *x* axis. The *y* axis represents the mRNA expression level of the *TNFAIP3* gene. Each dot represents the expression level of *TNFAIP3* for one individual; the individuals are grouped by genotype. (B) mRNA expression levels of *TNFAIP3* in unstimulated monocytes (No-Stim), LPS-stimulated monocytes, and Brucella-stimulated monocytes (Bru). (C) mRNA expression levels of *TNFAIP3* in monocytes with G/A and G/G genotypes. (D) mRNA expression levels of NF-κB1 in monocytes under resting, LPS-stimulated, and Brucella-stimulated conditions. * indicates a *P* value of <0.05; ** indicates a *P* value of <0.01.

To assess the role of the rs7749323 variant in regulating the expression of the *TNFAIP3* gene, we isolated PBMCs from 39 healthy individuals with different genotypes at the rs7749323 variant (13 G/G, 13 G/A, and 13 A/A) and determined the mRNA expression levels of the *TNFAIP3* gene using real-time quantitative PCR assays. In line with data from previously reported studies (25, 27), individuals carrying the protective A allele of rs7749323 displayed significant reductions in mRNA levels of the *TNFAIP3* gene (as shown in Fig. 3A). We further assessed the expression of NF-κB1 in monocytes with different genotypes at the rs7749323 variant. Our data showed that individuals carrying the protective A allele demonstrated a significant increase in NF-κB1 expression compared to that of carriers of the G allele (Fig. 3D). We further assessed mRNA expression levels of the *TNFAIP3* gene in monocytes stimulated with lipopolysaccharide (LPS) and B. abortus. As shown in Fig. 3B, the mRNA levels of *TNFAIP3* were significantly increased compared to those under unstimulated conditions. Interestingly, when samples were stratified by their genotypes at rs7749323, we observed that individuals carrying the G/G genotype are more sensitive to such stimulations than are individuals carrying the G/A genotype (Fig. 3C).

### The brucellosis-associated protective A allele significantly upregulates the expression of multiple cytokines in monocytes.

**FIG 4:**
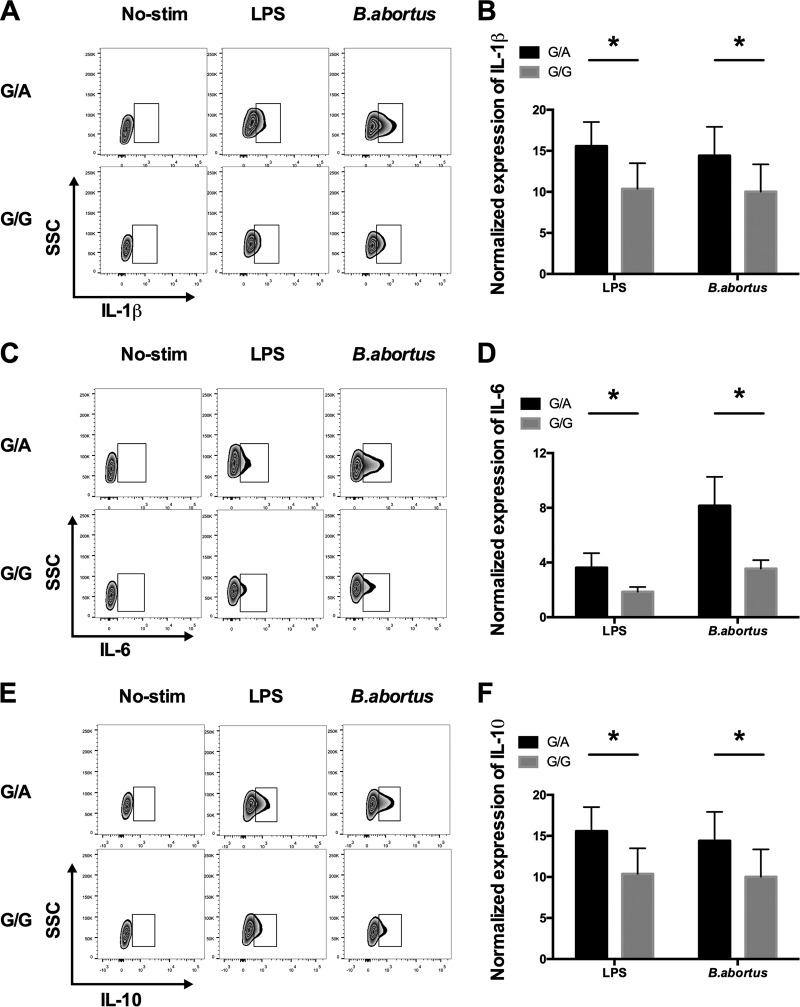
Protein expression levels of IL-1β, IL-6, and IL-10 are increased in B. abortus-stimulated monocytes from individuals carrying the protective A allele. Monocytes from 5 persons with the AG genotype and 5 persons with the GG genotype were separated and cultured in medium alone or with E. coli-derived LPS or heat-killed B. abortus for 12 h. The cells were then collected, and the protein levels of IL-1β (A), IL-6 (C), and IL-10 (E) were measured by intracellular staining; the IL-1β (B), IL-6 (D), and IL-10 (F) levels were higher for the TNFAIP3 AG genotype. * indicates a *P* value of <0.01. SSC, side scatter.

Our data showed that the protective A allele of the rs7749323 variant reduces the expression of *TNFAIP3* and increases the expression of NF-κB1. We further assessed expressions of multiple cytokines in monocytes with various genotypes at the rs7749323 variant. As shown in Fig. 4, we detected expressions of IL-1β, IL-6, and IL-10 in monocytes stimulated with E. coli-derived LPS and B. abortus. When comparing G/G and G/A genotypes, we observed that monocytes with the G/A genotype showed significant increases in the expression levels of IL-1β, IL-6, and IL-10. These data suggest that the brucellosis-associated protective A allele reduces the gene expression level of *TNFAIP3*, leading to the increased activity of NF-κB signaling and increased expression levels of multiple cytokines in monocytes.

## DISCUSSION

Increasing evidence shows that human diseases are influenced by genetic variants, but our understanding of the mechanisms that link a DNA sequence to a disease phenotype is limited. Large numbers of disease-susceptible nucleotide variants fall within the noncoding region of the human genome, making it likely that they act by modifying regulatory sequences for genes. In the present study, we investigated the TT>A functional variants, 42 kbp downstream of the *TNFAIP3* gene, in our cohort of 1,209 healthy controls and 150 brucellosis patients. To our knowledge, this is the first evidence that SLE-associated risk variants play a protective role in an infectious disease, namely, brucellosis.

Brucellosis is a well-known condition with importance in animal husbandry and in human disease in many regions of the world. Since Brucella species bacteria cause intracellular infection, the robust immune response against them is mediated by cell-mediated immunity, with a focus on macrophage activation (28). *TNFAIP3* encodes A20, a ubiquitin-editing enzyme with a pivotal role in negatively regulating NF-κB signaling downstream of multiple cell surface receptors (35). A20-deficient cells excrete high levels of proinflammatory cytokines and immediately activate lymphoid and myeloid cells. Genome-wide association studies (GWASs) of various autoimmune diseases have reported significant associations with sequence variants in the vicinity of the *TNFAIP3* gene (36). We previously described a pair of functional variants (TT>A) located in a distal enhancer of the *TNFAIP3* gene that were associated with SLE (25, 27). The enhancer element in which the TT>A variants are found binds to the NF-κB transcription factors p50, p65, and cREL and delivers them to the promoter region of the *TNFAIP3* gene, activating NF-κB-mediated transcription in multiple types of cells. The SLE-associated risk A allele disrupts the binding of NF-κB transcription factors and significantly reduces the expression of *TNFAIP3*. In our previously reported study, we also showed that TALEN-mediated TT>A enhancer knockout leads to reduced expression levels of *TNFAIP3* and significant increases in NF-κB signaling activity (26). These data have positioned the TT>A variants as NF-κB signaling modifiers, and they might play a role in infectious diseases. As shown in this study, the SLE-associated risk allele was significantly associated with a decreased risk of brucellosis.

Toll-like receptor (TLR) activation leads to intracellular signaling via MyD88 and IRAK-4, resulting in the activation of NF-κB and mitogen-activated protein kinases (MAPKs) and the production of inflammatory cytokines (37). The TT>A functional variants led to reduced expression levels of *TNFAIP3* and enhanced NF-κB signaling, which predisposes individuals to autoimmune diseases while protecting them from brucellosis. We therefore assessed the role of TT>A variants in regulating the gene expressions of multiple cytokines. Our data showed that the increased activity of NF-κB in monocytes results in significant increases in the expression levels of IL-1β, IL-6, and IL-10.

IL-1β is produced by monocytes, tissue macrophages, and dendritic cells (38). Increased levels of production of IL-1β were detected in the sera of untreated brucellosis patients and in the supernatants of Brucella suis-infected macrophage cell cultures, suggesting an important role of IL-1β in the pathogenesis of brucellosis (39, 40). After treatment, the level of IL-1β in the serum of brucellosis patients was significantly decreased and normalized (40). However, more studies are required to further understand the mechanism by which IL-1β influences brucellosis. Although the function of IL-6 in brucellosis is not clear, previous studies showed a significant association between an IL-6 polymorphism and brucellosis patients in Turkey (41, 42). Further evaluation of both the role of this IL-6 genetic variant and the function of IL-6 in brucellosis is required. IL-10 is a cytokine synthesis inhibitory factor because of its negative regulatory effect on cytokines, specifically IL-2 and gamma interferon (IFN-γ). In a murine model, IL-10 has been shown to influence the development of brucellosis by downregulating the response of CD4^+^ T helper cells and the secretion of IFN-γ (43, 44). On the other hand, IL-10 also promotes B cell antibody production. Furthermore, a genetic variant of the IL-10 gene has been reported to influence susceptibility to brucellosis (41, 42, 45, 46).

In addition to the TT>A polymorphic dinucleotide, the functional T>G (p.Phe127Cys) variant rs2230926 at exon 3 of *TNFAIP3* has been reported in different populations (47). Follow-up studies showed that the coding variant rs2230926 reduces the activity of A20, resulting in decreased NF-κB signaling (47). Further study of the role of this *TNFAIP3* gene coding variant in brucellosis is required for a better understanding of how functional genetic variants influence susceptibility to brucellosis.

In summary, we demonstrated for the first time that autoimmune disease-associated risk variants (TT>A) in the *TNFAIP3* gene play a protective role in brucellosis. The TT>A variants are associated with reduced expression levels of *TNFAIP3* and increased NF-κB activity. Functional studies revealed that individuals carrying the protective A allele display increased expression levels of multiple inflammatory cytokines.

## Supplementary Material

Supplemental material
