# Supplementary material for: An Autoimmune Disease-Associated Risk Variant in the TNFAIP3 Gene Plays a Protective Role in Brucellosis That Is Mediated by the NF-κB Signaling Pathway
Source: J Clin Microbiol. 2018 Mar 26;56(4):e01363-17. doi: 10.1128/JCM.01363-17 (PMC5869838; doi:10.1128/JCM.01363-17)
Supplement: Supplemental material [file supp_56_4_e01363-17__index.html]

An Autoimmune Disease-Associated Risk Variant in the TNFAIP3 Gene Plays a Protective Role in Brucellosis That Is Mediated by the NF-κB Signaling Pathway — Supplemental material 

# An Autoimmune Disease-Associated Risk Variant in the *TNFAIP3* Gene Plays a Protective Role in Brucellosis That Is Mediated by the NF-κB Signaling Pathway

## Supplemental material

- Supplemental file 1 -

  Tables S1 (The tag SNP rs7749323 is in LD with the TT>A variants), S2 (Demographics of the 150 cases and 1,209 independent controls), S3 (Primer and probe sequences designed for detection of rs7749323), and S4 (Primers used for real-time PCR analysis of gene expression)

  PDF, 75K
