# Supplementary material for: An Autoimmune Disease-Associated Risk Variant in the TNFAIP3 Gene Plays a Protective Role in Brucellosis That Is Mediated by the NF-κB Signaling Pathway
Source: J Clin Microbiol. 2018 Mar 26;56(4):e01363-17. doi: 10.1128/JCM.01363-17 (PMC5869838; doi:10.1128/JCM.01363-17)
Supplement: Supplemental material [file JCM.01363-17_zjm999095878s1.pdf]

Supplementary Table 1. The tag SNP rs7749323 is in LD with the TT>A variants

CHR_A	BP_A	SNP_A	CHR_B	BP_B	SNP_B	r^2
6	137908902	rs148314165	6	137908903	rs200820567	**1**
6	137908902	rs148314165	6	137909252	rs7749323	**1**
6	137908903	rs200820567	6	137909252	rs7749323	**1**

Supplementary Table 2. The demographics of the 150 cases and 1209 independent controls

	**Brucellosis**	**Control**	***P***
Age	45.94±10.90	47.17±10.61	0.128
≥30	138	1110	0.937
<30	12	99	
Gender			
Male	105	823	0.632
Female	45	386	
Total	150	1209	

Supplementary Table 3. Primer and probe sequences designed for detection of rs7749323

Oligonucleotide name	Oligonucleotide sequence（5′-3′）	Product size
rs7749323	PF: GAGATTCAGATTTAATTTGCCTAAGACA	85bp
	PR: TCTAGAGTGGTAGACCATGGAATCC	
	ATCGTGGATGGATG - FAM	
	TGATCGTGGATGGGTGT - VIC	

Supplementary Table 4. Primers used for real-time PCR analysis of gene expression

Gene	Primers	Product size
GAPDH	PF: 5′-CGGATTTGGTCGTATTGGG-3′	216bp
	PR: 5′-TCTCGCTCCTGGAAGATGG-3′	
TNFAIP3	PF: 5′-ATACCCCATTGTTCTCGGCTAT-3′	114bp
	PR: 5′-AATCTTCCCCGGTCTCTGTTAA-3′	
NF-κB1	PF: 5′-TGGGCTACACCGAAGCAAT-3′	69bp
	PR: 5′-GGGCCTGAGAGGTGGTCTT-3′	
